# Preventing Mammary Abscess

**Published:** 1879-10-20

**Authors:** Francis J. Shepherd

**Affiliations:** M. D., M. R. C. S., Eng.


### *A SIMPLE METHOD OF PREVENTING MAMMARY ABSCESS.*

BY FRANCIS J. SHEPHERD, M. D., M. R. C. S., ENG.

There is, I suppose, no accident which brings more discredit or gives more trouble to the surgeon than the occurrence in his practice of a "broken breast" case. My remedies (such as belladonna, hot oil frictions, etc.) have been advocated to prevent this painful affection, but I have found none more efficacious and speedy than the following simple plan which has been used for years with great success by old women in country parts; in fact, it may well be called, what indeed it is, an "old wife's remedy:" When the gland becomes indurated, painful, and has a glistening red look (symptoms, in fact, of approaching suppuration), take a large piece of ordinary sticking plaster and cut it a circular shape (a larger or smaller disc, according to the size of the affected breast); make a hole in the centre large enough to allow the nipple and half the areola to be seen, and apply this piece of plaster (after heating it) so that it will cover the *whole* breast, and that the nipple will protrude through the aperture in the centre. To make the plaster fit more accurately, its circumference should be deeply nicked at distances of about an inch. The plaster should be left on till the breast softens, or the plaster ceases to exercise even pressure. This simple method, in the half dozen cases I have seen it used, has acted magically, the breast softening and the pain disappearing in the course of twenty-four hours. In one case a woman, who had suffered on several previous occasions from broken breasts, came to the out-door department of the General Hospital with all the symptoms of fast approaching suppuration in her right breast; in fact, I considered that within twenty-four hours I should be obliged to use the knife. However, I said to the students that if there was anything in the plaster remedy, this would be a good case in which to try it. I applied the plaster in the way described above. Two days after, the woman returned and said, with a pleased smile, that it was the only remedy she had ever tried that had done her any good; that on previous occasions every remedy had failed to prevent her having a "broken breast." On examining the breast, I found it quite soft, painless, and with only one small lump of induration on the upper part, which disappeared in the course of a couple of days. In another case, where an abscess, due to depressed nipple, threatened, I applied the plaster as before, and in twenty-four hours there was hardly any induration, and no pain. In multiparæ, where the breast is dependent, in addition to covering the breast with plaster, I should advise supporting the breast by a band of plaster, 1½ inches broad, passing under the breast from shoulder to shoulder. I may say that I have only used this remedy in cases of threatened abscess, due to distension of milk ducts, depressed nipples, and obstruction to a free flow of milk, due to exposure of cold. I imagine the plaster acts simply by exercising an even pressure on the breast and giving support to it. I hope this method will be tried by some of your readers, and that they will give the results of their experience of it, beneficial or otherwise.—*Canada Medical and Surgical Journal*, July.

